# Advances in VNS efficiency and mechanisms of action on cognitive functions

**DOI:** 10.3389/fphys.2024.1452490

**Published:** 2024-10-09

**Authors:** Wendi Wang, Rui Li, Chuangtao Li, Qimin Liang, Xiaolin Gao

**Affiliations:** ^1^ Sports Rehabilitation Research Center, China Institute of Sport Science, Beijing, China; ^2^ School of Exercise Medicine and Health, Chengdu Sport University, Chengdu, China; ^3^ School of Physical Education and Sport Science, Fujian Normal University, Fujian, China; ^4^ School of Sport Science, Beijing Sport University, Beijing, China

**Keywords:** vagus nerve stimulation, cognitive enhancement, emotional processing, epilepsy, depression, Alzheimer’s disease, ADHD (attention deficit and hyperactivity disorder), long covid

## Abstract

**Objective:**

This systematic review aims to comprehensively analyze the efficacy and underlying mechanisms of vagus nerve stimulation (VNS) in enhancing cognitive functions and its therapeutic potential for various cognitive impairments. The review focuses on the impact of VNS on emotional processing, executive functions, learning, memory, and its clinical applications in conditions such as epilepsy, depression, Alzheimer’s disease, and other neurological disorders.

**Methods:**

A systematic search of electronic databases (PubMed, Scopus, Web of Science) was conducted using the keywords “vagus nerve stimulation,” “cognitive enhancement,” “emotional processing,” “executive function,” “learning and memory,” “epilepsy,” “depression,” “Alzheimer’s disease,” “neurological disorders,” “attention-deficit/hyperactivity disorder,” “sleep disorders,” and “long COVID.” The inclusion criteria encompassed controlled trials, longitudinal studies, and meta-analyses published in English between 2000 and July 2024.

**Results:**

A comprehensive review of 100 articles highlighted the cognitive effects of Vagus Nerve Stimulation (VNS). Studies show that VNS, especially through transcutaneous auricular VNS (taVNS), enhances emotional recognition, particularly for facial expressions, and improves selective attention under high cognitive demands. Additionally, VNS enhances learning and memory, including associative memory and spatial working memory tasks. In clinical applications, VNS exhibits promising benefits for improving cognitive functions in treatment-resistant epilepsy, depression, and Alzheimer’s disease.

**Conclusion:**

VNS represents a promising therapeutic approach for enhancing cognitive function across diverse patient populations. The reviewed evidence highlights its efficacy in modulating cognitive domains in healthy individuals and improving cognition in neurological conditions. However, the comparative effectiveness of different VNS modalities and the differential effects of online versus offline VNS on cognitive psychology require further investigation. Future research should focus on optimizing VNS protocols and elucidating specific cognitive domains that benefit most from VNS interventions. This ongoing exploration is essential for maximizing the therapeutic potential of VNS in clinical practice.

## 1 Introduction

Vagus nerve stimulation (VNS) is a type of neuromodulation therapy that targets excitability and changes the balance of autonomic nervous function (Ma et al., 2019; Jung et al., 2024). It does this by electrically stimulating the vagus nerve network. In 1985, Zabara 1985 first reported that VNS could suppress epileptic seizures in dogs. Since then, research on VNS for epilepsy has increased, revealing its positive effects on various diseases. In 1997 and 2005, the U.S. Food and Drug Administration (FDA) approved implantable VNS (iVNS) for the clinical treatment of drug-resistant epilepsy and depression. In 2017, the FDA also approved transcutaneous cervical VNS (taVNS) for migraines and cluster headaches. Currently, VNS can be classified into two main categories: invasive and non-invasive. Hese modalities have been studied in a range of neuropsychiatric disorders beyond epilepsy and depression, including Alzheimer’s disease, chronic pain, tinnitus, Parkinson’s disease, and post-stroke rehabilitation (detailed in Table 1). These applications highlight the versatility and potential of VNS as a therapeutic intervention across various conditions. For instance, studies have indicated its efficacy in reducing symptoms and improving the quality of life for patients suffering from these disorders. With the advancement of research, recent years have seen the emergence of new VNS stimulation patterns and protocols both domestically and internationally, with significant progress in the field of cognitive function regulation (Kalagara et al., 2024). This article aims to review the stimulation patterns, mechanisms, and effects of VNS on cognitive function, providing guidance and reference for clinical treatment and scientific research.

**TABLE 1:** VNS modalities across different disorders.

Pathology type	Non-invasive VNS	Invasive VNS
Depression	Liu et al. (2016); Fang et al. (2016); Bottomley et al. (2019)	George et al. (2000); Fang et al. (2016); Furmaga et al. (2012); Carreno and Frazer (2017); Desbeaumes Jodoin et al. (2018); Bottomley et al. (2019); Lespérance et al. (2024)
Epilepsy	Ghacibeh et al. (2006a); Lampros et al. (2021)	Clark et al. (1999); Aldenkamp et al. (2001); Dodrill and Morris. (2001); Sjögren, et al. (2002); Merrill et al. (2006); Danielsson et al. (2008); Marrosu, F. et al. (2003); Ghacibeh et al. (2006b); Orosz et al. (2014); Vaiman et al. (2017); Kimberley et al. (2018); Toffa et al. (2020); Jensen and Tsiropoulos (2024); Hamza et al. (2024); Winter et al. (2024)
Alzheimer’s Disease	Hachem et al. (2018); Caruso et al. (2018); Mertens et al. (2022)	Sjögren et al. (2002); Mertens et al. (2022); Jensen and Tsiropoulos (2024), Hamza et al. (2024)
Parkinson’s Disease	Farrand et al. (2017); Mondal et al. (2019), Mondal et al. (2021); Yu et al., 2021; Marano et al., 2024	Marano et al. (2024)
Pain	Napadow et al. (2012); Straube et al. (2015); Yuan et al. (2016); Garcia et al. (2017); Chakravarthy et al. (2015); Luo et al. (2020); Zeng et al. (2020); Zhang et al. (2019); Zhang et al. (2021b); Aranow et al. (2021)	Oshinsky et al. (2014); Chakravarthy et al. (2015)
Post-stroke Rehabilitation	Kalagara et al. (2024); Liu et al. (2024)	-
Insomnia	Zhang et al. (2021a)	-
Tinnitus	Yakunina et al. (2018); Lin et al. (2024)	Yakunina and Nam. (2021); Stegeman et al. (2021)
Other	Wang et al. (2021) (Alcohol Dependence); Zaehle and Krauel, (2021) (Attention-Deficit/Hyperactivity Disorder); Colzato et al. (2023) (Long COVID); Shi et al. (2024) (Functional Dyspepsia)	Aldenkamp et al. (2001) ADHD; Winter et al. (2024) (narcolepsy)

## 2 Modes

### 2.1 Classification of VNS

VNS is generally divided into four clinical forms. See Table 2 for details.

**TABLE 2:** Classification of VNS.

Type of VNS	Invasiveness	Electrode scheme	References
Cervically Implanted Vagus Nerve Stimulation (iVNS)	Invasive	Electrodes are surgically implanted at the cervical branch of the left vagus nerve, with the pulse generator placed subcutaneously at the upper chest near the pectoralis major muscle. This method is well-established in terms of efficacy and safety, but its high cost and risk of postoperative complications such as coughing, hoarseness, swallowing difficulties, and bradycardia limit its clinical use	Clark et al. (1998), Clark et al. (1999); Dodrill and Morris (2001); Aldenkamp et al. (2001); Sackeim et al. (2001); Sjögren et al. (2002); Danielsson et al. (2008); Merrill et al. (2006); Ghacibeh et al. (2006a), Ghacibeh et al. (2022); Klinkenberg et al. (2013); Orosz et al. (2014); Desbeaumes Jodoin et al. (2018); Toffa et al. (2020); Mertens et al. (2022)
Transcutaneous Cervical Vagus Nerve Stimulation	Non-invasive	Electrodes are placed on the neck surface using a portable handheld device to deliver electrical currents. Stronger currents are required to penetrate the skin barrier, which can frequently activate other cervical nerves and vagal efferent fibers. Common adverse effects include tingling at the stimulation site, neck pain, dizziness, headaches, and oropharyngeal pain	Krahl et al. (1998); Dorr and Debonnel (2006); Raedt et al. (2011); Clancy (2014); Landau et al. (2015); Liu et al. (2016); Redgrave et al. (2018); Burger and Verkuil (2018); Sclocco et al. (2019); Farmer et al. (2021)
Transcutaneous auricular vagus nerve stimulation	Non-invasive	Surface electrodes are placed on the auricle, typically in the cymba concha area, to stimulate the auricular branch of the vagus nerve (ABVN). The ABVN’s afferent fibers project to the nucleus of the solitary tract (NTS) in the brainstem, regulating neural pathways. This method generates a relatively small electric field intensity in the ear area, resulting in minimal adverse effects. However, its clinical efficacy remains somewhat controversial	Steenbergen et al. (2015, 2020); Straube et al. (2015); Chen et al. (2015); Jacobs et al. (2015); Liu et al. (2016); Fang et al. (2016); Beste et al. (2016); Badran et al. (2018); Keute et al. (2018a); Redgrave et al. (2018); Burger and Verkuil. (2018); Jongkees et al. (2018); Sellaro et al. (2018); Yakunina et al. (2017), Yakunina et al. (2018), Yakunina and Nam. (2021); Keute et al. (2019), Keute et al. (2021); Zhang et al. (2019), Zhang et al. (2021b); Mertens et al. (2020); Toffa et al. (2020); Luo et al. (2020); Maraver et al. (2020); Borges. et al. (2020); Lampros et al. (2021); Sun et al. (2021); Wang et al. (2021); Aranow et al. (2021); Yu et al. (2021); D'Agostini et al.(2022); Zhang et al. (2021a); D'Agostini et al.(2022)
Percutaneous auricular vagus nerve stimulation (paVNS)	Minimally invasive	This involves using 2-3 micro-needle electrodes to penetrate the skin of the ear area and stimulate the ABVN. Compared to surface electrodes, needle electrodes are smaller, allowing for more precise spatial positioning in the ear area. However, the minimally invasive nature of paVNS presents challenges regarding patient acceptance, including discomfort and risk of minor skin infections	Kovacic et al., ((2017)

### 2.2 Methods of VNS

Conventional VNS typically follows an open-loop stimulation model, where the stimulation parameters are pre-set before treatment and do not change in response to neural activity during treatment. Clark et al. (1998), Clark et al. (1999) discovered that cervical iVNS in rats and epilepsy patients follow an intensity-dependent inverted U-shaped dose-effect relationship, where moderate current intensity yields better memory performance than lower or higher current intensities. Buell et al. (2018) recorded auditory cortex activity in rats via EEG and verified that VNS-induced cortical plasticity follows an inverted U-shaped function of VNS pulse frequency.

This phenomenon can be explained by the Yerkes-Dodson law: for simpler tasks, individual responses increase linearly with motivational stimuli; however, as task difficulty increases, the optimal response threshold for stimuli decreases (Yerkes and Dodson, 1908; Broadhurst, 1957; Calabrese, 2008a; Calabrese, 2008b). In tcVNS studies, reported stimulation parameters vary widely, and subject responses lack consistency across studies. This inconsistency may result from individual differences and methodological heterogeneity, such as the innervation patterns of the ABVN in the auricle (Yakunina et al., 2017; Burger and Verkuil, 2018), pre-stimulation vagal tone (Clancy, 2014), respiratory cycles (Sclocco et al., 2019), and stimulation sites. Some studies suggest that taVNS has consistent effects regardless of the stimulation site (left or right ear) or ear area (cymba concha or lobule) (Chen et al., 2015; Keute et al., 2021). Despite the lack of a standardized percutaneous VNS protocol, the “International Consensus on Minimum Reporting Standards for Transcutaneous Vagus Nerve Stimulation Studies (2020)" (Farmer et al., 2021) recommends standardizing commonly used stimulation parameters in tcVNS studies, including stimulation site, electrodes, duty cycle, frequency (Hz), intensity (mA), pulse width (μs), and waveform.

Closed-loop VNS involves continuously measuring subjects’ behavioral performance, brain activity, and peripheral physiological indicators during stimulation, dynamically adjusting stimulation parameters based on the subjects’ state. A team from Harvard Medical School developed the Respiratory-gated Auricular Vagal Afferent Nerve Stimulation (RAVANS) method, which uses a pressure sensor placed on the subject’s chest to deliver taVNS stimulation during the expiratory/inspiratory phase based on chest expansion. Results show that stimulation during exhalation (eRAVANS) enhances treatment efficacy, potentially because the ventral respiratory group (VRG) in the medulla sends excitatory signals to the NTS during exhalation, optimizing VNS stimulation (Napadow et al., 2012; Garcia et al., 2017; Sclocco et al., 2019). Besides RAVANS, Cook et al. designed a myoelectric-triggered auricular VNS system (MAAVNS) based on orofacial muscle movement to improve patients’ swallowing function (Cook et al., 2020). Thanks to advancements in wireless technology, wireless EEG, ECG, and subcutaneous fluid signal devices (SHS) are increasingly used in closed-loop taVNS systems (Yu et al., 2021). However, eliminating motion artifacts in closed-loop VNS and ensuring its sustainability and stimulation efficacy remain research priorities for the future.

## 3 Mechanisms of central regulation by VNS

### 3.1 Afferent network of the vagus nerve

The afferent branches of the vagus nerve can be functionally divided into: 1) General somatic afferent fibers (GSA) originating from the superior ganglion, transmitting general sensations from the posterior wall of the external auditory canal and the outer surface of the tympanic membrane, terminating in the spinal trigeminal nucleus;2) General visceral afferent fibers (GVA) originating from the inferior ganglion, distributed to the pharynx, larynx, trachea, lungs, esophagus, abdominal viscera, aortic pressure, chemoreceptors, and the dura mater of the posterior cranial fossa, terminating in the caudal part of the nucleus tractus solitarius (NTS); 3) Special visceral afferent fibers (SVA) originating from the inferior ganglion, receiving taste information from the epiglottis area, terminating in the rostral part of the NTS (Ruffoli et al., 2011). Approximately 80% of vagus nerve fibers are sensory afferent fibers (George et al., 2000), primarily transmitting general somatic and visceral sensations. The NTS is the main hub of the vagus nerve’s afferent network, where most afferent fibers terminate. For example, the NTS sends these fibers to the locus coeruleus (LC), the dorsal raphe nucleus (DRN), and the parabrachial nucleus (PBN). There are close neural connections between brainstem nuclei and the thalamus, hippocampus, and amygdala, regulating perception, learning, memory, and emotional functions. Furthermore, connections are established through the nucleus basalis and cingulate cortex with the prefrontal cortex (PFC), orbital frontal cortex (OFC), and sensorimotor cortex, which participate in the regulation of cortical and subcortical circuit excitability (Hachem et al., 2018).

### 3.2 Central mechanisms of VNS

#### 3.2.1 Inhibition of neuroinflammatory response

Neuroinflammation, linked to neurovascular unit (NVU) damage, microglia and astrocyte activation, and increased blood-brain barrier (BBB) permeability, can be mitigated by VNS, which helps maintain BBB integrity (Chen et al., 2018). This has a “neuroprotective” effect and is used to treat neurological and psychiatric disorders (Borovikova et al., 2000; Shytle et al., 2004; Varatharaj and Galea, 2017; Fonseca et al., 2019). The classic anti-inflammatory mechanism of VNS is the “cholinergic anti-inflammatory pathway” (CAP). A study by Borovikova et al. (2000) found that acetylcholine (ACh) released after VNS can lower the levels of cytokines that cause inflammation (TNF-α, IL-1β, IL-6, and IL-8) that are caused by lipopolysaccharide (LPS) without changing the levels of cytokines that stop inflammatory responses (IL-10, IL-4, and TGF-β). In 2012, Olofsson et al., 2012 studied the CAP pathway in more detail and discovered that norepinephrine (NE), which is released when the vagus nerve stimulates the celiac ganglion and splenic nerve, can increase the number of T lymphocytes and ACh release through β2-adrenergic receptors (β2-AR). ACh can connect to α7-nicotinic acetylcholine receptors (α7nAChR), which turns on macrophages in the spleen and lowers the production of TNF-α. In the same way, Shytle et al. (2004) discovered that VNS can turn on microglial α7nAChR, which stops the CAP pathway from making pro-inflammatory cytokines. Kaczmarczyk et al. (2018) said that VNS can change the microglial phenotype from neurodestructive to neuroprotective. This is done by increasing the release of BDNF, bFGF, and anti-inflammatory factors while decreasing the release of pro-inflammatory factors. This protects the neurons. Besides that, VNS can turn on specific mAChR central muscarinic acetylcholine receptors. These can then activate vagal efferent fibers and stop the growth of inflammation (Pavlov et al., 2009). Additionally, mAChR is involved in hippocampal theta rhythm modulation, which is crucial for learning, memory, and anxiety regulation (Broncel et al., 2018). Increased BBB permeability is another critical factor in neuroinflammation, with NVU damage being the core mechanism. The NVU is made up of vascular endothelial cells, smooth muscle cells, cholinergic and adrenergic nerve terminals, astrocytes, and perivascular cells (microglia, macrophages, and mast cells). It is very important for keeping the brain’s microenvironment stable and regulating blood flow, BBB substance exchange, immune surveillance, nutritional support, and coagulation balance (Iadecola, 2010; Kalaria, 2010). Researchers have discovered that increasing the release of LC and NE can turn on endothelial cell α7nAChR, which makes them better at pinocytosis (Kimura et al., 2019). Excitation of the vagus nerve can stop microglia and reactive astrocyte activation (Chen et al., 2018; Yang et al., 2018), stop aquaporin-4 (AQP-4), lower TNF-α levels, and improve tight junction protein protection of the BBB (Lopez et al., 2012), which stops neurodegenerative changes caused by inflammation (Varatharaj and Galea, 2017). Also, dorsal motor nuclei of the vagus (DMV) and paraventricular nuclei of the hypothalamus (PVN) that are activated by the vagus nerve help release growth hormone-releasing peptides (Ghrelin) and oxytocin, which help control inflammation and protect the brain-blood barrier (Bansal et al., 2012; Yuan et al., 2016; Collden et al., 2017; Panaro et al., 2020).

Along with its impact on immune cytokines and BBB permeability, VNS may also help reduce inflammation by controlling gut microbiota and improving cerebrospinal fluid (CSF) exchange in the glymphatic system (Bohórquez et al., 2015; Bonaz et al., 2019; Cheng et al., 2020; Zhang et al., 2020), though the exact ways it does this need more research.

#### 3.2.2 Promoting neurogenic signaling pathways

VNS promotes the release of neurotransmitters and chemical molecules in the brain. These are the main ones: the NE pathway, the 5-HT pathway, the dopamine (DA) pathway, the brain-derived neurotrophic factor-tyrosine kinase B (TrkB) pathway, and the -aminobutyric acid (GABA) pathway.

The neurotransmitter GABA is mostly found in the medial septum (MS), the nucleus ambiguus (NA), the dorsal motor nucleus of the vagus (DMV), and the nucleus tractus solitarius (NTS) (Bennett et al., 1987; Helm et al., 2005; Herman et al., 2009; Pelkey et al., 2017). It is an inhibitory neurotransmitter. Herman et al. (2009) discovered that stimulating NTS can increase GABA signaling and send it to DMN and NA, making epileptic seizures less severe. Marrosu et al. (2003) found that GABA can control cortical excitability through GABAA receptors on the cerebral cortex to help people with epilepsy. And Keute et al. (2018a), Keute et al. (2018b) discovered that VNS can also act on GABAergic neurons in the motor cortex to control autonomic behavior inhibition. Furthermore, it has been discovered that GABAA and GABAB receptors in MS help control changes in the hippocampus after VNS stimulation. This can impact how people deal with anxiety and their ability to learn new things (Broncel et al., 2019). It can be seen that VNS stimulation can promote GABAergic pathways to regulate abnormal brain excitation and achieve comprehensive protective effects.

Noradrenaline (NE) is an excitatory neurotransmitter, and the locus coeruleus (LC) is the most abundant region of noradrenergic neurons in the brain. Short-term and long-term VNS can raise the firing rate of LC neurons and keep them active for a long time, causing NE to build up in the prefrontal lobe, amygdala, and hippocampus (Groves, Bowman and Brown, 2005; Hulsey et al., 2017). The rise in NE is strongly connected to cognitive function and memory performance (Ciampa et al., 2022). Some scholars have observed that after VNS, there is also an increase in NE levels in the medial and thalamic areas, as well as other cerebral cortex areas (Landau et al., 2015). The LC, the core node of the noradrenergic pathway, can sustain damage in experiments. VNS-mediated antiepileptic effects are blocked by LC (Krahl et al., 1998; Raedt et al., 2011; Liu et al., 2016); additionally, LC mediates the release of 5-HT and dopamine (DA) (Ruffoli et al., 2011).

The dorsal raphe nucleus (DRN) is where the VNS controls the 5-HT pathway. Stimulating the VNS can raise the level of 5-HT in the DRN and hippocampus, which can help people with depression (Furmaga et al., 2011). At the moment, there is disagreement about whether NTS and DRN connect directly through fibers. However, electrophysiological studies have shown that NTS can be sent through LC to indirectly control DRN (Dorr and Debonnel, 2006). They found that the discharge rate of LC increased after long-term and short-term VNS, and the discharge rate of DRN increased significantly only after long-term VNS. A study by Manta et al. (2009) found that the increasing DRN discharge frequency stopped happening after selective inhibition of LC-mediated norepinephrine neurons. This means that 5-hydroxytryptamine and norepinephrine work together and have a purpose in the brain. Manta et al. (2013) and Farrand et al. (2017) also revealed dopamine (DA) in the midbrain ventral tegmentum, substantia nigra, striatum, subventricular nucleus, and frontal lobe.

Brain-derived neurotrophic factor (BDNF) can prevent neuronal death and is an important hippocampal plasticity neurotrophic factor (Hofer and Barde, 1988; Zhao et al., 2007). A study by Furmaga et al. (2012) and Carreno and Frazer (2014) shows that long-term VNS can change hippocampal neurons through the aTnAChR mechanism, raise BDNF levels, improve mouse memory, and boost hippocampal tyrosine kinase B receptor B (AAkt) and cellular external signals controlled by S6K and CAMP response element binding protein (CREP), a group of cytokines.

## 4 VNS and its effects on cognitive function

### 4.1 VNS’s role in cognitive processing

VNS is most commonly studied in the cognitive domain of emotional functioning. VNS can enhance subjects’ ability to recognize emotions in others’ facial expressions. Ventura-Bort et al. (2021) required subjects to complete memory tasks while receiving taVNS stimulation and conducted a recognition test 1 week later. The results showed that the taVNS group performed better on emotional images compared to the sham group, with no significant difference in performance on neutral images between the two groups. Colzato et al. (2017) found that taVNS only improved performance on simple tasks of the Reading the Mind in the Eyes Test (RMET), with no significant effect on complex RMET tasks. Sellaro et al. (2018), based on the above studies, further explored the differential effects of taVNS on facial and body emotion recognition, showing that subjects’ scores in facial emotion recognition improved without affecting body emotion recognition. Sellaro therefore suggests that taVNS can enhance the brain’s ability to recognize emotions but is sensitive only to prominent stimuli that enhance attention, such as faces and eyes. Maraver et al. (2020) discovered that people who received taVNS were more accurate in tasks that came after direct gaze stimuli. This improvement happened regardless of the emotion (anger, fear) or the time between tasks, which supports Sellaro’s findings even more. Additionally, VNS can regulate participants’ inner motivation and evaluation before and after task completion, with delayed satisfaction being an important characteristic of self-emotional control.

Researchers have found that the impact of taVNS on delayed reward discount rates is dependent on the individual’s positive emotional level, reflecting the effectiveness of taVNS in emotional control (Steenbergen et al., 2020). De Smet et al. (2021) found that after subjects receiving taVNS were required to re-evaluate pictures previously rated negatively, their intense emotions were significantly reduced compared to the control group. Neuser et al. (2020), Ferstl et al. (2021) did one of the most important studies on emotional control. They found that taVNS significantly increased participants’ positive emotions after they worked hard to complete low-reward tasks. They also found that lower baseline positive emotions were significantly related to better taVNS effects. This study suggests that taVNS can encourage participants to work harder under low-value rewards to enhance their behavioral motivation, which is highly significant for improving patient behavioral motivation in clinical rehabilitation settings.

VNS also has a significant impact on executive attention functions, particularly selective attention and cognitive control. Steenbergen et al. (2015) used the stop-change paradigm to test people’s selective attention function. They discovered that taVNS improved people’s ability to choose their responses during action cascade processes, which means they had faster reactions when doing two behaviors right after each other. In more research on inhibitory control, Beste et al. (2016) discovered that taVNS did not improve performance in the reverse inhibition paradigm but did significantly improve performance in response inhibition paradigms involving task loads. Jongkees et al. (2018) also discovered that subjects receiving taVNS did not show any return inhibition phenomenon in continuous response tasks with high cognitive loads, suggesting that taVNS can improve cognitive selection processes when high-selectivity requirements are present. However, its effect on cognitive control is limited under conditions of lower loads or involvement of working memory. Borges et al. (2020) partly corroborated these findings, minimizing working memory load and using the Flanker test and Stroop questionnaire to test subjects’ inhibitory control capabilities. The results showed that under taVNS stimulation, there was no improvement in subjects’ inhibitory control performance, but there was a significant improvement in their performance in tasks involving task switching. Colzato looks at the vagus nerve network’s GABAergic pathway and the locus coeruleus-norepinephrine (LC-NE) pathway and says that taVNS can stimulate the GABAergic pathway in the cortex to promote inhibition. This weakens competitive selection in tasks that need high selectivity and makes it easier for people to choose between competing options. Network reset theory says that taVNS turns on the LC-NE after a global attention reset to change people’s behavior and stop them from investing too much time in tasks (Colzato et al., 2018a; Colzato et al., 2018b).

Learning and memory are core functions in cognitive processing and are closely related to emotional and attentional domains. In 2015, Jacobs et al. found that VNS can enhance elderly associative memory for faces, while some studies have shown that taVNS has no significant effect on vocabulary recognition memory (Mertens et al., 2020). One possible explanation for this phenomenon is that taVNS drives subjects to allocate more attentional resources to targets related to faces and emotional cues through its enhanced emotional effects, thereby reinforcing the encoding and consolidation stages of memory. Research from the past has shown that the amygdala controls explicit emotion and declarative memory in the hippocampus. VNS can stimulate these areas (Badran et al., 2018; Singh et al., 2022). It is worth noting that Sun et al. (2021) found that taVNS significantly improved subjects’ performance on spatial working memory tasks using the n-Back paradigm, possibly related to taVNS activation of the prefrontal lobe, but the neuroimaging mechanisms require further study.

### 4.2 The effects of VNS on patients with cognitive impairments

#### 4.2.1 VNS and Epilepsy

The cognitive improvement effects of VNS in patients were first reported by Clark et al. (1999). They found that moderate current intensity (0.5 mA) VNS had the best effect on language memory improvement in epilepsy patients, whereas higher intensities (0.75–1.5 mA) led to reduced memory performance. Dodrill and Morris (2001) divided epilepsy patients into high and low stimulation groups and found no significant improvement in cognitive function after 12–16 weeks of follow-up with iVNS, although the high stimulation group experienced fewer emotional and physiological problems compared to the low stimulation group. Ghacibeh et al. (2006a), Ghacibeh et al. (2006b) used the Hopkins Verbal Learning Test (HVLT) to study 10 epilepsy patients implanted with iVNS. The results showed that there was no significant improvement in the learning part of the HVLT. However, the recognition and recall scores were significantly higher than those in the sham group. This suggests that VNS improves the encoding-consolidation phase of short-term memory in people with epilepsy. However, Ghacibeh also found a certain degree of decline in creativity and cognitive flexibility in epilepsy patients after receiving iVNS. A study by Mertens et al. (2022) compared the effectiveness of iVNS and taVNS in epilepsy patients. They discovered that acute iVNS and taVNS had no effect on language memory, but chronic iVNS over 6 weeks improved patients’ immediate recall and delayed recognition. This suggests that cumulative effects are one potential mechanism of VNS for cognitive impairment therapy.

In addition to adult epilepsy, many scholars have studied the effects of VNS on cognitive improvement in pediatric epilepsy patients, but the results are still controversial (Aldenkamp et al., 2001; Danielsson et al., 2008; Klinkenberg et al., 2013; Orosz et al., 2014). The cognitive-functional efficacy of VNS in patients with depression has also been studied to some extent. Unlike epilepsy patients, Sackeim et al. (2001) found no significant correlation between cognitive improvement in patients with major depressive disorder and VNS current intensity. Fourteen depressed patients who were implanted with VNS were studied for 2 years. It was found that improvements in their depressive symptoms were strongly linked to their attention and visuospatial working memory (Desbeaumes Jodoin et al., 2018). It is inferred that VNS may indirectly improve cognitive function by alleviating patients’ depressive symptoms.

#### 4.2.2 VNS and Alzheimer’s disease

Two-thirds of dementia cases are diagnosed with Alzheimer’s disease (AD), characterized by neuronal deposition of amyloid-β plaques and neurofibrillary tau tangles, inflammatory activation of glia, reduced synaptic capacity, and neuronal loss. The Sjogren team conducted a series of studies on whether iVNS could improve cognitive status in patients with AD. In 2002, Sjogren et al. reported that 6 months after VNS implantation, seven out of 10 AD patients showed improvement in ADAS-cog scores, while nine out of 10 patients showed improvement in MMSE scores. A subsequent extended study of 17 AD patients after iVNS surgery found a median decrease of 4.8% in tau protein in cerebrospinal fluid within 1 year, while phosphorylated tau protein increased by 5%, providing new evidence for the physiological mechanisms of VNS in cognitive improvement (Merrill et al., 2006).

Aside from tau protein levels, stress appears to be another significant factor influencing AD (Caruso et al., 2018). Research has found elevated cortisol levels in biological fluids such as plasma, saliva, and cerebrospinal fluid in AD patients, which can exacerbate disease progression. Chronic VNS has been shown to effectively reduce serum cortisol levels, potentially improving the prognosis for AD patients by mitigating stress severity (O’Keane et al., 2005). While VNS can reduce cortisol and thereby delay the progression of AD, research on the regulation of the hypothalamic-pituitary-adrenal (HPA) axis in AD patients remains limited. Understanding how VNS regulates cortisol and its mechanisms to limit the negative impact of stress on individuals with brain diseases is crucial.

Epel et al. (2004) found that psychological stress is associated with telomere shortening. Currently, there is significant research discussing the relationship between telomere length and AD. Most studies suggest that shorter telomeres are associated with an increased risk of AD (Hackenhaar et al., 2021). Previous case-control studies and meta-analyses have shown the presence of short leukocyte telomere length (LTL) in individuals diagnosed with AD (Boccardi et al., 2020). Short baseline LTL is associated with a higher risk of developing AD (Koh et al., 2020) and all-cause dementia (Honig et al., 2012), although some studies have found no association between LTL and AD (Hinterberger et al., 2017). Notably, another longitudinal time-to-event analysis study found a nonlinear relationship between LTL and AD, with both short and long LTL associated with an increased risk of AD (Fani et al., 2020). Similar short and long-term LTL risk correlations have been observed for amnestic mild cognitive impairment (aMCI), considered a prodromal stage of AD (Roberts et al., 2014).

Telomere dysfunction induced by damage can occur regardless of telomere length (Brandr, 2019). According to the neuro-immune-senescence integrative model (NISIM; Ask and Sütterlin, 2022), reduced vagal regulation capacity represents decreased splenic vagal input and increased inflammation. NISIM posits that the prefrontal cortex (PFC) influences splenic inflammation levels when exerting regulatory effects on arousal through the vagus nerve. Research by Torvald et al. suggests that the activity of the HPA axis is related to telomere length, possibly indicating that HPA axis dysregulation leads to increased inflammation levels and subsequent ROS-induced telomere damage. This provides preliminary evidence for the potential clinical intervention of vagus nerve stimulation in improving telomere dysfunction and reducing peripheral inflammation levels.

Overall, VNS emerges as a promising intervention for AD, targeting cognitive impairments through modulation of tau proteins and addressing inflammatory and stress-related pathways through cortisol and telomere regulation. Further research is necessary to elucidate the precise mechanisms and optimize VNS protocols for maximum therapeutic benefit.

#### 4.2.3 VNS and Sleep

VNS has become an intervention for sleep disorders (Zhang S. et al., 2021). Srinivasan et al. (2023) investigated the effects of taVNS on sleep disorders exacerbated in elderly healthcare workers. The results showed that taVNS significantly improved sleep quality and reduced anxiety. taVNS may improve sleep quality by modulating the brain’s default mode network (DMN) and salience network (SN). The DMN, active during rest, is involved in self-relevant thoughts and introspection, with its dysfunction associated with mental health issues such as anxiety and depression, which in turn affect sleep quality. The SN is responsible for processing significant sensory information and helps shift attention from internal thoughts to external stimuli, maintaining good mood and sleep. Additionally, brain-derived neurotrophic factor (BDNF) plays a crucial role in promoting neuroplasticity and recovery. Increased levels of BDNF are associated with improved mood, cognitive function, and sleep quality. taVNS may support brain health and enhance sleep quality by increasing plasma BDNF levels.

Meanwhile, Werner et al. (2015) examined the relationship between cardiac vagal control (CVC) and sleep quality in healthy women, finding that higher levels of CVC, measured by high-frequency heart rate variability (HF-HRV), were associated with better sleep quality. This suggests that good autonomic regulation and higher CVC can improve sleep quarters.

The arousal and wake-promoting effects of VNS have been demonstrated in animal studies and are well-known side effects of VNS treatment for epilepsy and depression. Winter et al. (2024) suggested that VNS may be a promising non-drug treatment for narcolepsy. Moreover, VNS may further stabilize neural networks and improve sleep quality during sleep, reducing the risk of seizures (Vespa et al., 2021). However, research on VNS for sleep disorders is still limited, and further high-quality randomized controlled trials are needed to verify its efficacy.

#### 4.2.4 VNS and other diseases

Furthermore, studies have found that VNS has certain therapeutic effects on alcohol dependence (Wang et al., 2021) and COVID-19-related symptoms. Recent studies, including those by Azabou et al. (2021), have highlighted the potential of VNS to modulate the immune response in COVID-19 patients. This modulation is believed to occur via CAP activation, leading to reduced levels of pro-inflammatory cytokines such as TNF-α, IL-1β, and IL-6. They found that VNS significantly reduced pro-inflammatory cytokine levels, suggesting a potential mechanism for alleviating the hyperinflammatory state often seen in severe COVID-19 cases.

In another study, Colzato et al. (2023) demonstrated that long COVID, characterized by persistent symptoms such as “brain fog,” anxiety, depression, and cognitive deficits, is believed to be associated with brainstem dysfunction and disrupted vagal signaling. Studies suggest that taVNS may help ameliorate these symptoms by enhancing vagal activity and directly activating brainstem nuclei involved in cognitive and affective regulation. Also, taVNS is a non-pharmacological intervention that can be self-administered, making it a practical and accessible treatment option. Its safety profile is favorable, with minimal adverse effects reported compared to other invasive vagus nerve stimulation methods.

Expanding on these findings, Zheng et al. (2024) conducted a pilot study on the efficacy of taVNS in a female cohort with Long COVID. The study included 24 female patients who underwent a 10-day t-VNS intervention. Results demonstrated significant improvements in cognitive functions, anxiety, depression, and sleep post-intervention, with sustained benefits observed at a 1-month follow-up. However, olfactory performance did not show significant improvement, indicating the need for further investigation into this specific symptom.

Despite of novel findings in researches, several limitations need to be addressed. First, the studies reviewed often have small sample sizes and lack diversity in patient demographics, particularly the study by Zheng et al. (2024), which focused exclusively on female patients. This limits the generalizability of the results to the broader population. Also, the variability in VNS protocols, such as stimulation parameters and duration, complicates the comparison of outcomes across studies. Moreover, the pilot nature of these studies means that long-term safety and efficacy data are limited. The studies primarily report short-term benefits, and there is a need for longitudinal research to determine the sustained impact of VNS on COVID-19-related symptoms. At the same time, potential side effects should not be ignored (Mastitskaya et al., 2021), such as voice alteration, cough, dyspnea, dysphagia, etc.

## 5 Conclusion

VNS as a neuroregulatory technique holds promising applications in both clinical practice and research. As more clinical trials are done, more expert opinions are published, and non-invasive stimulation devices and closed-loop feedback stimulation technology get better, the use of VNS is moving toward more standardized and varied growth. VNS has been proven effective in modulating various cognitive domains in healthy individuals, and it shows potential benefits in improving cognition in treatment-resistant epilepsy, depression, Alzheimer’s disease, addictive disorders, and sleep disorders. However, there is currently a lack of comparative studies between different VNS stimulation modalities. Moreover, the differential effects of online VNS versus offline VNS on cognitive psychology remain unclear. Existing clinical trials often provide broad assessments of cognitive functions in patients without detailed scrutiny of various cognitive processing stages. To address these gaps, future research should delve deeper into these areas.

Overall, VNS represents a promising avenue for enhancing cognitive function across different patient populations. Continued research efforts are crucial to elucidating the optimal protocols, mechanisms of action, and specific cognitive domains that can benefit most from VNS interventions.
